# Impact of repetitive transcranial magnetic stimulation on cortical activity: a systematic review and meta-analysis utilizing functional near-infrared spectroscopy evaluation

**DOI:** 10.1186/s12984-024-01407-9

**Published:** 2024-06-24

**Authors:** Shao-Yu Chen, Meng-Hsuan Tsou, Kuan-Yu Chen, Yan-Ci Liu, Meng-Ting Lin

**Affiliations:** 1 Department of Physical Medicine and Rehabilitation, National Taiwan University Hospital, College of Medicine, National Taiwan University, No. 7 Chung-Shan South Road, Taipei City, 10002 Taiwan; 2 School and Graduate Institute of Physical Therapy, College of Medicine, National Taiwan University, 3F., No.17, Xuzhou Rd., Zhongzheng Dist, Taipei City, 10002 Taiwan; 3 Physical Therapy Center, National Taiwan University Hospital, College of Medicine, National Taiwan University, No. 1, Changde St., Zhongzheng Dist, Taipei City, 10022 Taiwan

**Keywords:** Repeated transcranial magnetic stimulation (rTMS), Functional near-infrared spectroscopy (fNIRS), Primary motor cortex, Cortical hemodynamics, Cortical excitability

## Abstract

**Background:**

Repeated transcranial magnetic stimulation (rTMS) could induce alterations in cortical excitability and promote neuroplasticity. To precisely quantify these effects, functional near-infrared spectroscopy (fNIRS), an optical neuroimaging modality adept at detecting changes in cortical hemodynamic responses, has been employed concurrently alongside rTMS to measure and tailor the impact of diverse rTMS protocols on the brain cortex.

**Objective:**

This systematic review and meta-analysis aimed to elucidate the effects of rTMS on cortical hemodynamic responses over the primary motor cortex (M1) as detected by fNIRS.

**Methods:**

Original articles that utilized rTMS to stimulate the M1 cortex in combination with fNIRS for the assessment of cortical activity were systematically searched across the PubMed, Embase, and Scopus databases. The search encompassed records from the inception of these databases up until April, 2024. The assessment for risk of bias was also conducted. A meta-analysis was also conducted in studies with extractable raw data.

**Results:**

Among 312 studies, 14 articles were eligible for qualitative review. 7 studies were eligible for meta-analysis. A variety of rTMS protocols was employed on M1 cortex. In inhibitory rTMS, multiple studies observed a reduction in the concentration of oxygenated hemoglobin [HbO] at the ipsilateral M1, contrasted by an elevation at the contralateral M1. Meta-analysis also corroborated this consistent trend. Nevertheless, certain investigations unveiled diminished [HbO] in bilateral M1. Several studies also depicted intricate inhibitory or excitatory interplay among distinct cortical regions.

**Conclusion:**

Diverse rTMS protocols led to varied patterns of cortical activity detected by fNIRS. Meta-analysis revealed a trend of increasing [HbO] in the contralateral cortices and decreasing [HbO] in the ipsilateral cortices following low frequency inhibitory rTMS. However, due to the heterogeneity between studies, further research is necessary to comprehensively understand rTMS-induced alterations in brain activity.

## Introduction

Repeated transcranial magnetic stimulation (rTMS), a magnetic field-generating device, induces currents across the superficial cerebral cortex to achieve neural modulation effects, thereby enhancing cortical excitability or instigating inhibition [1, 2]. Beyond its local impact on cortical excitability at the site of stimulation, rTMS exerts influence over contralateral brain areas through intricate reciprocal inhibitory projections and complex neuronal networks [3, 4]. Moreover, the effects of intracortical facilitation or inhibition induced by rTMS endure for a considerable duration even after the cessation of stimulation [5, 6].

rTMS has garnered widespread application in clinical contexts, particularly in treating conditions such as major depressive disorder, neuropathic pain, and aiding motor recovery in post-stroke patients [7]. A variety of rTMS protocols have very different neuromodulation effects on the primary motor cortex. Studies indicate that frequencies below 1 Hz, known as low-frequency stimulation, inhibit cortical excitability, while frequencies above 5 Hz or even 10 Hz, referred to as high-frequency stimulation, excite the brain [8]. Quadripulse stimulation (QPS) consists of four monophasic pulses in a single stimulation burst. QPS with a short inter-pulse interval potentiates cortical excitability while long-interval QPS elicits depressive effects [9]. Theta burst stimulation (TBS), a relatively new technique, utilizes high-frequency bursts at 50 Hz combined with clusters of stimuli delivered at a rate of 5 bursts per second. This approach significantly reduces administration time while producing effects comparable to traditional high and low-frequency stimulation methods, with continuous TBS (cTBS) exerts inhibitory effects on the motor cortex, while intermittent TBS (iTBS) achieves excitatory effects [10].

rTMS targeting the primary motor cortex (M1) was predominantly investigated in previous studies. Prior studies have demonstrated that the application of rTMS to the M1 in healthy human subjects resulted in measurable changes in cortical excitability, as evidenced by alterations in motor-evoked potentials (MEPs), resting motor threshold (RMT) intensities, and other intracortical neural activities [11, 12]. To quantify the impact of rTMS on the cortex, a series of investigations have been undertaken on healthy individuals. These studies concurrently employed rTMS along with electroencephalography (EEG) [13, 14], positron emission tomography (PET) [3, 15], functional magnetic resonance imaging (fMRI) [16, 17], and functional near-infrared spectroscopy (fNIRS) to quantify neural activity [18]. fNIRS is an optical neuroimaging modality using near-infrared spectroscopy to discern alterations in cortical hemodynamic responses as a result of neural activity within the superficial cerebral cortex [19]. The utilization of fNIRS alongside rTMS has several advantages, encompassing its minimal susceptibility to electromagnetic interference from rTMS pulses, thereby yielding diminished measurement artifacts [20]. fNIRS is capable of assessing changes in brain activity during dynamic functional tasks, and affords flexibility in the placement of rTMS coils during data acquisition. The portability of fNIRS, its commercial availability, and affordability further bolster its appeal.

Numerous investigations have combined rTMS in conjunction with fNIRS over the motor cortex to evaluate its impact on healthy human subjects. A previous review comprising nine such studies predominantly adopted a low-frequency inhibitory rTMS protocol [21]. In recent years, new studies featuring a larger number of participants, more rigorous experimental designs, and the incorporation of diverse rTMS protocols, including high-frequency facilitative protocols, have been conducted [22–24].

Therefore, our systematic review and meta-analysis seeks to provide a contemporary update on the utility of fNIRS in capturing alterations in cortical activity over the M1 cortex among healthy individuals during and post rTMS. This review comprehensively addresses the effects observed during and after rTMS sessions, encompassing periods of both rest and task performance. Moreover, the diverse effect of different rTMS protocols is thoroughly examined within the framework of this review.

## Materials and methods

This systematic review was conducted in accordance with the recommendation of Preferred Reporting Items for Systematic Reviews and Meta-Analyses (PRISMA) 2020 version. PubMed, Embase, and Scopus were searched for English-written, peer-reviewed articles from the earliest records to April, 2024. The following search terms (“fNIRS” OR “NIRS” OR “Near infrared spectroscopy” OR “Near-infrared spectroscopy” OR “Optical topography” OR “Diffuse optical tomography” OR “DOT”) AND (“rTMS” OR “TMS” OR “Transcranial Magnetic Stimulation”) AND (“motor cortex” OR “motor hotspot” OR “Brodmann area 4” OR “M1”) were utilized.

### Eligibility criteria

All original studies including randomized control trials, crossover studies, and observational studies were selected, but not case reports or conference papers. The inclusion criteria were (1) studies conducted on healthy subjects of any age or gender without any past history of neurological or psychiatric diseases; (2) studies utilizing rTMS to stimulate the M1 cortex combined with fNIRS to evaluate cortical activity. We excluded articles containing the following: (1) studies that used single-pulse TMS, since there were no known therapeutic or long-lasting effects to cortical activities; (2) studies where the stimulation targeted cortices other than the M1, for example, the prefrontal or the primary sensory cortex.

### Selection process

Two medical researchers independently assessed the titles and abstracts of all studies retrieved from the aforementioned databases. Resolution of any disagreements regarding the suitability of particular studies was achieved through deliberative discourse, leading to a unanimous consensus. Subsequently, a single researcher conducted a comprehensive review of the complete texts of the selected articles for final inclusion. Each included article underwent a meticulous reevaluation by all authors.

### Risk of bias assessment

Risk of bias was assessed using a revised Cochrane risk-of-bias tool for randomized trials (RoB 2.0) on one randomized controlled trial [25]. Other observational studies were assessed by using The Newcastle-Ottawa Scale (NOS) [26]. Evaluation of bias risk was executed independently by two authors involved in the review process. Discordance was resolved through discussion and consensus. If consensus could not be attained, the corresponding authors served as the final arbiter.

### Meta-analysis

A total of 7 research [22, 23, 27–31] with extractable raw data were eligible for meta-analysis. Required information including number of subjects, mean and standard deviation of different parameters were extracted from the forementioned studies and analyzed with RevMan.

## Results

### Characteristics of included studies

**Fig. 1:**
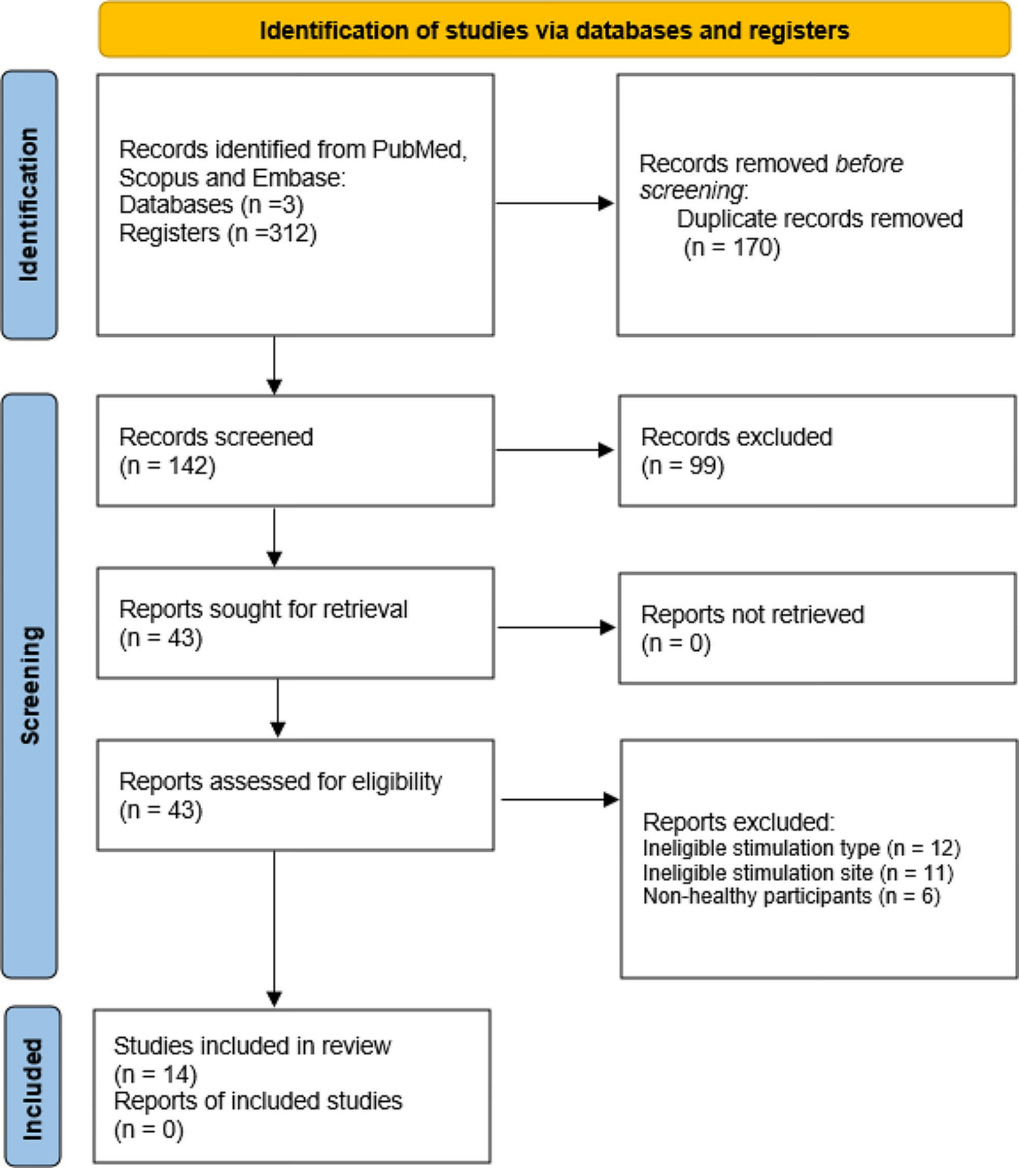
The PRISMA 2020 flow diagram for the systematic review, depicting the database searched, the number of abstract screened, full-text reviewed or excluded

A total of 312 studies were identified through the database search. After removing 170 duplicated records, 142 articles were screened for titles and abstracts. Subsequently, 43 articles were reviewed for eligibility, and eventually, 14 studies were included in our qualitative review (Fig. 1).

Among these 14 studies, one of them was a double-blinded randomized controlled trial (RCT) [24], while the rest of the studies were observational studies including cross-over trials, cohort studies, and some without controlled groups [22, 23, 27–37]. All studies enrolled healthy adults. The stimulation of rTMS was applied to the M1 cortex in all studies, but with different parameter settings and protocols. Ten studies utilized traditional rTMS [22–24, 27, 30–34, 36], while two studies used QPS [29, 35], and three studies used iTBS or cTBS [24, 28, 37]. Cortical activities of the ipsilateral, contralateral, or bilateral cortex were recorded using fNIRS, represented by a combination of cerebral blood flow, hemoglobin concentration [Hb], oxygenated hemoglobin concentration [HbO], and deoxygenated hemoglobin concentration [HbD] during or after rTMS stimulation. Neuronal activation is typically correlates with an increase in [HbO] and a comparatively modest decrease in [HbD], in accordance with neurovascular coupling. Furthermore, [Hb] is derived from the summation of [HbO] and [HbD], serving as a marker for vasomotor activity [38–40]. The Twelve studies recorded the on-line effect simultaneously during rTMS stimulation [22–24, 27–31, 33–36], while eleven studies measured the cerebral hemodynamics after stimulation [23, 24, 27–33, 36, 37]. In most studies, the participants were at rest, except for two studies that involved a finger-tapping task [32] and a serial reaction time task using the non-dominant hand [23], respectively.

### Quality of the included studies

Critical appraisal was conducted employing RoB 2.0 for randomized studies and NOS for non-randomized observational studies (Table 1). The double-blind RCT by Li et al. [20] exhibited a low risk of bias following RoB 2.0 assessment. Among the thirteen observational studies, nine were rated as good quality, while four were deemed to be of poor quality based on NOS assessment due to absence of a control group and poor comparability of cohorts based on control for confounders.

### fNIRS measurements during and after rTMS

The 14 studies included in this systematic review assessed changes in cortical hemodynamics during and after rTMS (Table 2). Nine articles [23, 24, 27–31, 33, 36] assessed changes both during and after stimulation, three [22, 34, 35] assessed changes during stimulation, and two [32, 37] only assessed cortical changes after rTMS stimulation.

fNIRS using different numbers of emitters, receivers, and channels, has been employed to assess corresponding cortical activation changes by detecting increase or decrease in [Hb], [HbO], and [HbD] levels (Table 2). Hada et al. assessed fNIRS responses in the left M1 after application of four different parameters of rTMS to the same region where all conditions resulted in a decrease in [HbO] in the stimulated region [27]. Kozel et al. and Tian et al. both applied 1 Hz rTMS to the left M1, and found a significant [HbO] decrease in the ispsilateral (left) M1 region during stimulation [30, 33]. Groiss et al. observed significant decreases in [HbO] in the left M1 after application of two different conditions of quadripulse stimulation (QPS), QPS-5 and QPS-50, to the same region [35].

### Meta-analysis of eligible studies

**Fig. 2:**
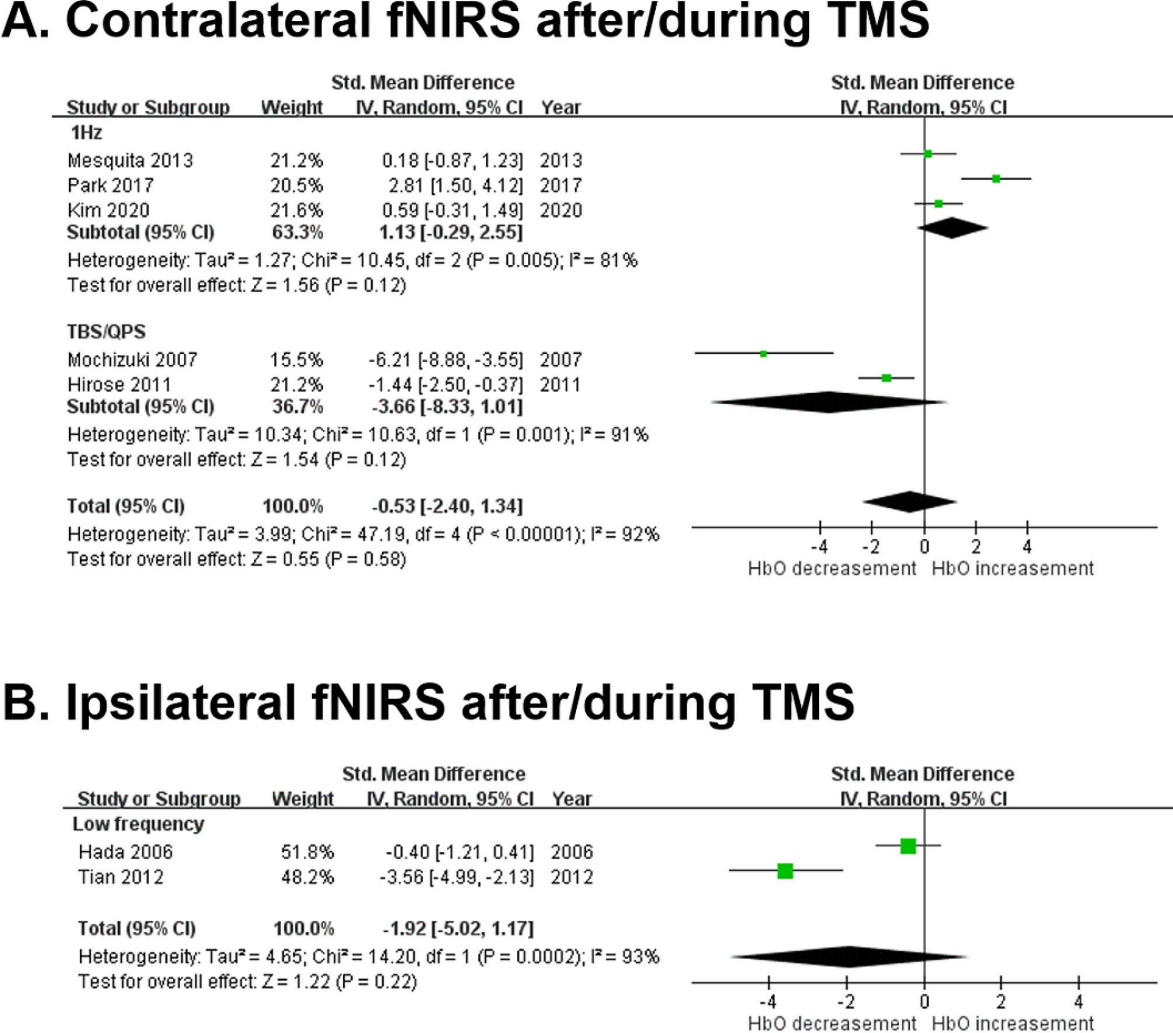
The meta-analysis demonstrated no statistically significance difference of HbO change in either contralateral or ipsilateral cortices during or after rTMS compared to baseline

A total of 7 studies were eligible for meta-analysis [22, 23, 27–31]. Five studies utilized contralateral fNIRS to assess cortical excitability after or during TMS [23, 28–31], while two papers utilized ipsilateral fNIRS for the same purpose [22, 23, 27–31]. In the contralateral fNIRS measurements for cortical excitability (Fig. 2A), there was no significant difference before and after TMS regarding HbO change. Subgroup analysis (Fig. 2A) revealed similar results with no significant HbO difference, but there appeared to be a trend indicating that 1 Hz TMS tended to increase contralateral HbO, whereas TBS or QPS tended to decrease contralateral HbO. In the ipsilateral fNIRS measurements for cortical excitability (Fig. 2B), there was also no significant difference before and after TMS regarding HbO change. High heterogeneity existed in all meta-analyses, indicating significant variability between studies.

However, there is a trend of increased [HbO] in the contralateral cortices (Fig. 2A) and decreased [HbO] in the ipsilateral cortices in the low frequency (1 Hz) inhibitory rTMS group (Fig. 2B). TBS and QPS also tended to decrease [HbO] in contralateral cortices.

**Table 1:** Assessing the risk of bias of included studies using the Newcastle-Ottawa Scale (NOS)

	Selection				Comparability	Outcome			Quality
Study	Representative-ness of the exposed cohort	Selection of the non-exposed cohort	Ascertainment of exposure	Outcome was not present at start of study	Comparability of cohorts based on control for confounders	Assessment of outcome	Was follow-up long enough for outcomes to occur	Adequacy of follow-up of cohorts	
Hada et al., 2006	★	★	★	★	★★	-	★	★	Good
Chiang et al., 2007	★	★	★	★	★★	-	★	★	Good
Mochizuki et al., 2007	★	★	★	★	★★	-	★	★	Good
Kozel et at., 2009	★	-	★	★	-	-	★	★	Poor
Hirose et al., 2011	★	★	★	★	★★	-	★	★	Good
Nasi et al., 2011	★	★	★	★	★★	-	★	★	Good
Tian et al., 2012	★	-	★	★	-	-	★	★	Poor
Groiss et al., 2013	★	★	★	★	★★	-	★	★	Good
Mesquita et al., 2013	★	-	★	★	-	-	★	★	Poor
Park et al., 2017	★	★	★	★	★★	-	★	★	Good
Li et al., 2019	★	-	★	★	-	-	★	★	Poor
Kim et al., 2020	★	★	★	★	★★	-	★	★	Good
Gorban et al., 2023	★	★	★	★	★★	-	★	★	Good

**Table 2:** Effects of rTMS stimulation to M1 on fNIRS measurement in healthy subjects

References	Study design	No. of subjects	Stimulation parameters	Sham stimulation	Stimulation area	fNIRS specifications	fNIRS measurement area	Timing of measurement	Task	Findings
Hada et al., 2006	Single-blind, within-subject design	12 right-handed healthy subjects	rTMS, 10 times (1)120% RMT, 2 Hz (2)120% RMT, 0.5 Hz (3) 80% RMT, 2 Hz (4) 80% RMT, 0.5 Hz	N/A	Left M1	OMN-2001 Shimadzu Corp. Japan (A pair of NIRS probes attached to the rTMS coil)	Left M1	During rTMS	Rest	- Decreased [Hb] [HbO], increased [HbD] at the ipsilateral M1 in all groups. - Mildly increased [Hb] and [HbO] after 5s of rTMS at the ipsilateral M1 in the 120% RMT groups.
								After rTMS (for 3 min)	Rest	- Greatest peaks of [Hb] [HbO] decrement and [HbD] increment just after rTMS
Chiang et al., 2007	Single-blind, within-subject design	5 right-handed healthy subjects	(1) rTMS, 115% RMT, 1 Hz for 20 min (2) Sham	A non-discharging coil and a real stimulation placed at 90 degrees	Right M1	NIRO-200 monitor Hamamatsu Photonics KK	Left M1	After rTMS (for 115 min, intermittent)	Finger tapping task	- Increased [HbO] at the contralateral M1, for 40 min. - Decreased [HbD] at the contralateral M1, for 15 min.
Mochizuki et al., 2007	Single-blind, within-subject design	8 right-handed healthy subjects	(1) iTBS, 80 or 100% AMT. triple-pulse burst 50 Hz* 10 bursts, 30 pulses (2) Sham	A non-discharging coil at the stimulation site a real stimulation 10 cm above the head	Left PM, M1 or S1	ETG-A1; Hitachi Medical Corp. Japan (4 pairs of emitters and detectors)	Right prefrontal cortex, PM, M1 and S1	During rTMS	Rest	- iTBS over M1 elicited decreased [HbO] at the contralateral M1.
Kozel et at., 2009	Observational	11 healthy subjects	rTMS, 120% RMT, 1 Hz, 10 pulses* 15 trains * 2 sessions	N/A	Left M1 then the left PM	CW5, TechEn Inc, USA (8 sources and 16 detectors)	Bilateral M1 then PM	During rTMS	Rest	- Decreased [HbO] at the bilateral M1, with less decrement at the ipsilateral versus the contralateral M1.
Hirose et al., 2011	Single-blind, within-subject design	9 right-handed healthy subjects	QPS, 110% AMT, 24 bursts (1) QPS-5: ISI = 5 ms (2) QPS-50: ISI = 50 ms (3) Sham	A non-discharging coil at the stimulation site, and a real stimulation 10 cm above the head	Left M1	ETG-4000; Hitachi Medical Corp., Japan (5 pairs of emitters and detectors)	Right M1, PM and S1	During QPS	Rest	- Decreased [HbO] at contralateral M1, PM and S1 in QPS-5 vs. sham. - Decreased [HbO] at contralateral M1 in QPS-50 vs. sham.
Nasi et al., 2011	Observational, controlled	10 healthy subjects	rTMS, 75% RMT, at 0.5, 1, and 2 Hz in 8s * 25 trains	N/A	Left M1 (Control group: Left shoulder)	2 sources and 6 detectors	Bilateral M1	During rTMS	Rest	- Decreased [Hb] (mostly [HbO]) at the bilateral M1 in the 2 Hz group.
Tian et al., 2012	Observational (test-retest reliability assessment)	11 healthy subjects	rTMS, 120% RMT, 1 Hz, 10 pulses* 15 trains * 2 sessions	N/A	Left M1 then the left PM	CW5, TechEn Inc., USA (8 sources and 16 detectors)	Bilateral M1 then PM	During rTMS	Rest	- Decreased [HbO] at the bilateral M1 and PM with a moderate to high reliability.
Groiss et al., 2013	Single-blind, within-subject design	10 healthy subjects	QPS, 90% AMT, 0.2 Hz (1) 24 bursts * 3 trains of QPS-5 or QPS-50 (2) 10 bursts of QPS-5 and QPS-50 each (3) Sham	Sham stimulation with ISI = 5 ms (SHAM-5) or 50 ms (SHAM-50)	Left M1	ETG-4000; Hitachi Medical Corp., Japan (5 pairs of emitters and detectors, 24 channels)	Left M1, S1, PM, SMA and PFC.	During QPS	Rest	- Decreased [HbO] during QPS-5 at the ipsilateral M1 and PM compared to baseline level and sham.
								After QPS (for 55–180 s)	Rest	- [HbO] decrement mentioned above peaked at 10s after QPS-5 and returned to baseline level at 20s after QPS-5.
Mesquita et al., 2013	Observational	7 right-handed healthy subjects	rTMS, 95% RMT, 1 Hz, totally 1200 pulses	N/A	Left M1	Self-designed diffuse correlation/ optical spectroscopy	Bilateral M1	During rTMS	Rest	- Increased CBF and [HbO] at the ipsilateral M1. No change at the contralateral M1.
								After rTMS (for 4 min)	Rest	- Increased CBF and [HbO] at the ipsilateral M1 compared to baseline levels.
Park et al., 2017	Single-blind, within-subject design	10 right-handed healthy subjects	(1) rTMS, 90% RMT, 1 Hz, totally 1200 pulses (2) Sham	A disconnected figure-of-eight coil	Left M1	OMM-3000; Shimadzu, Japan. (8 pairs of emitters and detectors)	Right M1 and PM	During rTMS	Rest	- Increased [HbO] at the contralateral M1, decreased [HbD] at the contralateral M1 and PM compared to sham.
								After rTMS (for 20 min)	Rest	- The above changes lasted for at least 20 min after rTMS compared to baseline levels.
Li et al., 2019	Observational	20 right-handed healthy subjects	rTMS, 90% RMT, 10 Hz, 40 trains (each train 2s stimulation + 28s rest)	N/A	Bilateral PFC, PM, M1, middle PFC and SMA	NIRx Medical Technologies, USA (16 emitters and 15 detectors, 46 channels)	Where the rTMS stimulation was	During rTMS	Rest	- Decreased [HbO] and HbO AUC at the bilateral M1 with gradual recovery. - Decreased functional connectivity within prefrontal areas as well as between symmetrical M1-pairs.
								After rTMS	Rest	- The above [HbO] and HbO AUC decrements returned to baseline levels within 5 min.
Kim et al., 2020	Single-blind, crossover design	10 right-handed healthy subjects	(1) rTMS, 100% RMT, 1 Hz, totally 1200 pulses (2) Sham	Diverting the coil 90 degrees with the same stimulation	Left M1 HK, Left M1 hMHS	NIR Scout ^®^; NIRx Medical Technology, Germany. (8 sources and 16 detectors, 31 channels)	Right M1	During and after rTMS	Serial reaction time task	- Increased integral value of HbO at the contralateral M1 after hMHS-rTMS compared to baseline.
Li et al., 2022	Double-blind, RCT	40 right-handed healthy subjects	80% RMT (1) pcTBS: 50 Hz, 3 pulses* 400 times (2) iTBS: 50 Hz, 3 pulses * 10 times (3) rTMS: 10 Hz, 10 s * 15 trains (4) Sham	Present but not defined	Left M1	BS-3000, Wuhan Znion Technology Co., Ltd., China. (12 pairs of emitters and detectors, 37 channels)	Bilateral prefrontal area	Right after rTMS	Pain Tolerance Threshold (PTT)	- Increased [HbO] at the bilateral DLPFC in the pcTBS and iTBS groups when compared to other certain groups.
								60 min after rTMS	PTT	- Increased [HbO] at the bilateral DLPFC and FPC in the pcTBS and 10 Hz rTMS groups compared to other certain groups. - Increased [HbO] at the ipsilateral DLPFC compared to baseline in the pcTBS group.
Gorban et al., 2023	Single-blind, within-subject design	10 right-handed healthy subjects	80% AMT (1) cTBS: 50 Hz of 3 pulses, total 600 pulses (2) iTBS: 2 s trains of TBS, total 600 pulses (3) Sham	Active coil tilted 90°	Left M1 (hand region)	Frequency-Domain Multi-Distance NIRS, Imagent, ISS, Champaign IL, USA	Left M1 (hand region)	After rTMS	Evoked change after a single-pulse TMS (100% RMT) & sham stimulus	- Increased [Hb] after cTBS compared to baseline - A trend of increased [HbO] change after cTBS, but not statistically significant.

AMT = active motor threshold, AUC = area under curve, CBF = cerebral blood flow, cTBS = continuous theta-burst stimulation, DLPFC = dorsolateral prefrontal cortex, FPC = frontopolar cortex, HbD = deoxygenated hemoglobin, HbO = oxygenated hemoglobin, HK = anatomical hand knob, hMHS = hand motor hotspot, ISI = interstimulus interval, iTBS = intermittent theta-burst stimulation, M1 = primary motor cortex, MT = motor threshold, pcTBS = prolonged continuous theta-burst stimulation, PFC = prefrontal cortex, PM = premotor cortex, QPS = quadripulse stimulation, RCT = randomized controlled trial, RMT = resting motor threshold, S1 = primary sensory area, SMA = Supplementary Motor Area

### Effects of rTMS with fNIRS measurements during functional tasks

Two of the thirteen studies performed fNIRS during functional tasks after rTMS. Chiang et al. examined the effects of 1 Hz rTMS to the right M1 on fNIRS measurements with right finger-tapping task, revealing an increase of [HbO] in the contralateral M1 and lasting for 40 min after the stimulation [32]. In the study by Kim et al., 1 Hz of rTMS was applied to different target sites, the anatomical hand knob region (HK) and the hand motor hotspot region (hMHS), of the left M1 during a serial finger-tapping task, showing significantly increased changes of [HbO] in the contralateral right M1 region after application of rTMS to the hMHS [23].

### rTMS parameter settings on cortical hemodynamics

Various rTMS protocols, such as low-frequency rTMS, high-frequency rTMS, iTBS, cTBS, and QPS, have been applied to the M1 cortex of healthy subjects. Among the thirteen studies reviewed, low-frequency rTMS was applied in eight studies [23, 27, 30–34, 36], high frequency rTMS in two studies [22, 24], both short and long intervals of QPS (QPS-5, QPS-50) in two studies [29, 35], and cTBS/iTBS in three studies [24, 28, 37]. As for the rTMS intensity, submaximal stimulation (75–95% RMT/AMT) was implemented in nine studies and maximal or supramaximal stimulation (100–120% RMT) in seven studies. During rTMS protocols that suppressed cortical excitability such as low-frequency rTMS, a decrease in ipsilateral [HbO] with increase of [HbO] on the contralateral side was generally observed. On the contrary, facilitating rTMS protocols such as iTBS elicited an increased [HbO] on the ipsilateral side [24] or a decreased [HbO] in the contralateral cortex [28]. However, when implementing 10 Hz high-frequency rTMS [22], a global decrease in [HbO] over multiple motor and sensory cortical areas as well as functional connectivity between these cortices was observed. This decrement of [HbO] returned to baseline levels gradually during rTMS. In terms of QPS, both facilitating QPS-5 and inhibitory QPS-50 elicited decreased [HbO] in the bilateral cortex during stimulation [29, 35] with QPS-5 exhibiting a more significant effect. Regarding cTBS, despite its inhibitory traits, recent studies have demonstrated increasing cerebral blood flow and [HbO] changes over the ipsilateral or bilateral cortices when evoked by a finger tapping task or a single-pulse TMS after cTBS. [24, 37]

## Discussion

This systematic review and meta-analysis investigated the impacts of rTMS on cortical hemodynamics in healthy adults, wherein fNIRS was used in to monitor hemodynamic activity. Because of the varied study protocol designs and stimulation parameters of rTMS, disparate patterns of fNIRS measurement were observed among included studies. Nevertheless, significant changes in cortical hemodynamics following rTMS to the M1 cortex were indicative of alterations in neuronal activity and blood flow in the stimulated and contralateral brain regions. Our study contributes valuable insights into the effects of rTMS on brain hemodynamic changes and highlights the need for further investigation to elucidate the underlying mechanisms.

### fNIRS measurements during and after rTMS stimulation

Several studies revealed that a decrease in [HbO] was generally found in the M1 region of the stimulated side. Our meta-analysis further supports this trend, showing a decrement in [HbO] on the stimulated side following low-frequency rTMS. However, the activation patterns observed on the contralateral side exhibit considerable variability across studies. The significant decrease in ipsilateral [HbO] may be associated with vasoconstriction at the ipsilateral side of stimulation [34]. Magnetic stimulation creates a local electric field in the cerebral tissue that leads to increased activity of cerebral neurons and contraction of smooth muscle of the walls of the stimulated cerebral blood vessels, leading to decreased blood volume as reflected by decreased concentration of hemoglobin recorded by fNIRS [34]. Decreased [HbO] during rTMS stimulation indicates inhibition in the stimulated cortex that is consistent with physiological neurovascular coupling [35]. Post-excitatory inhibition exhibited by neurons after an initial stimulation reflects the changes in electrophysiological properties of the cells, which may explain the decrease in [HbO] recorded by fNIRS [35].

Furthermore, contrasting [HbO] responses are observed in the contralateral motor cortex, shedding light on the interhemispheric interactions following rTMS stimulation. In the cortical region contralateral to the stimulated side, an increase in [HbO] is observed when 1 Hz of rTMS was applied to the right M1, and this effect continued as increased [HbO] was found to last for 40 min post-stimulation [32]. Park et al. observed significant [HbO] increases in the contralateral M1 during and after rTMS stimulation, with changes lasting 20 min post-stimulation [31]. Kim et al. applied 1 Hz of rTMS and found an increases in [HbO] of contralateral M1, with significantly greater increases in the motor hot spot area [23]. The deactivation of the stimulated cortex was typically coupled with excitable contralateral cortex during rTMS, indicating the effects of interhemispheric modulation [23, 32].

In contrast, several studies found significant decreases in [HbO] in contralateral M1 regions [28, 29] during and after facilitator rTMS application. In Li et al.’s study, a significant reduction in [HbO] and decreased inter-regional connectivity were observed during high frequency rTMS (10 Hz) [22]. Though high frequency rTMS is thought to induce facilitatory effects on the stimulated region through the mechanisms of interhemispheric inhibition, this may in turn induce an inhibitory effect on the contralateral regions. Hirose et al. observed that both QPS-5 and QPS-50 (considered as facilitator) induced a decreased [HbO] in contralateral M1 shortly after onset, returning to baseline within 2 to 3 min [29]. Similarly, Mochizuki et al. reported significant decreases in [HbO] in the contralateral M1 during 30 pulses iTBS [28]. Our meta-analysis further corroborated these findings, demonstrating a consistent trend wherein inhibitory rTMS led to an increase in [HbO] in the contralateral M1 region, while facilitatory rTMS induced a decrease in [HbO] in the same region. These opposing [HbO] patterns suggest the presence of dense mutual interactions between the motor cortices between hemispheres. Activation of the ipsilateral motor cortex may trigger a reciprocal suppressive effect on its contralateral counterpart, highlighting the intricate interplay between hemispheres in response to rTMS stimulation [29].

### Effects of rTMS stimulation on fNIRS measurements during functional tasks

As rTMS has the ability to modulate cortical excitability, in recent years, there has been a greater focus on the effects of rTMS on task performance and the associated changes in neural activity. Consistent with the concept of brain lateralization, the M1 contralateral to the moving finger during finger-tapping exhibited increases in [HbO] due to the high oxygen demand of the task [32]. This increased excitability of the non-stimulated cortex further supports the concept of interhemispheric inhibition between the motor cortices during active movement [41, 42]. Furthermore, inhibition of the target site may produce a secondary effect distant from the stimulation target site due to transcallosal connections between cortices [43]. Thus, functional tasks during rTMS application may serve to amplify hemodynamic changes, specifically increases in [HbO] to the hemisphere contralateral to the moving limb. Further studies elucidating the effects of rTMS on task-evoked fNIRS activity are needed to better inform clinical models where functional tasks are executed during rTMS stimulation.

### Effects of different rTMS parameter settings on cortical hemodynamics

It is well-established that rTMS produces frequency-dependent changes in cortical excitability when applied over the M1 region [31, 44]. High frequency rTMS increases cortical excitability, whereas low frequency rTMS reduces excitability [24].

Interestingly, 1 Hz stimulation may have different effects on the M1 cortex of healthy individuals depending on whether the stimulation is continuous or separated into trains. A continuous 1 Hz rTMS elicited increased [HbO] and decreased [HbD] at the contralateral M1 in several controlled studies [23, 31, 32], indicating interhemispheric interactions of motor cortices discussed in previous sections. On the contrary, Kozel et al. and Tian et al. from the same research group utilized 1-Hz rTMS with a “10s stimulation time” to “80s rest time” on-off ratio [30, 33]. A decreased [HbO] at the bilateral M1 after intermittent 1 Hz stimulation was observed, suggesting decreased cortical activities in both hemispheres. This draws a stark contrast compared to stroke patients, in which both continuous [45, 46] and intermittent trains [47–49] of 1 Hz rTMS posed an inhibitory effect on the stimulated M1 and excitatory effect on the opposite side. Several factors may come into play leading to the above contradicting results. First of all, the two studies employing intermittent 1 Hz rTMS exhibit certain instrumental limitations [30, 33]. In order to accommodate the fNIRS system, the distance between the rTMS coil and cortex was increased. As mentioned by the authors, this led to an inadequate stimulation power (less than 120% RMT as originally planned) and difficulties determining RMT in some patients, and may greatly influence parameters detected by fNIRS. Secondly, the sample size in studies involving healthy subjects is significantly smaller compared to those involving stroke patients. Hence, future research may be warranted to explore the different effects of intermittent versus continuous 1 Hz rTMS on fNIRS parameters in the motor cortices of healthy subjects.

TBS is another paradigm encompassing bursts of high-frequency stimulation, wherein cTBS yields an inhibitory effect, and iTBS a facilitatory effect on MEP amplitudes [6, 50]. QPS is a relatively novel rTMS protocol comprised of a series of four consecutive stimulation pulses in rapid succession. Notably, this protocol yields a facilitation effect on MEPs when implemented with short intervals, such as 5 milliseconds. Conversely, the protocol exerts a suppressive influence with longer intervals [51, 52]. These rTMS protocols also possess the capacity to induce enduring after-effects on neural plasticity and excitability through long-term potentiation or depression (LTP/LTD) after the stimulation ceases [51].

### Configuration of fNIRS and rTMS with potential interference

Depending on the site of rTMS stimulation and site of fNIRS measurements, the placement of the respective devices may vary from study to study with potential interference, thus affecting the observed results of cortical hemodynamic changes. In studies where rTMS application and fNIRS measurements were obtained in the same hemisphere or in bilateral hemispheres, the distance of the rTMS coil was adjusted to accommodate the placement of the fNIRS optodes, as coils in close proximity may introduce mechanical noise into the signals or cause displacement of an optode entirely [21]. In such cases, the coil is placed above the fNIRS montage and farther from the scalp, and increasing power is adjusted for the rTMS to account for weakening of the magnetic field with distance [21]. The increased power of the rTMS may introduce extra artifacts into the fNIRS measurements, such as facial muscle twitching [31]. Special consideration must be taken during data analyses to ensure that transient artifacts induced by rTMS are removed.

### Limitations

Although this systematic review is one of the first to explore changes in cortical hemodynamics using fNIRs after rTMS application, it has not without limitations. First, the included articles were mainly preliminary observational studies with small sample sizes (ranging from 5 to 20 participants, only one study [24] recruited 40 participants). The results therefore may only reflect a limited population of healthy adults. Second, although fNIRS was used to monitor changes in either unilateral or bilateral hemispheres, very few channels were identified, which may limit the extent of the M1 region observed. Most of the articles provided little to no specification about the standards of optode placement, and did not adhere by the International 10–20 or 10 − 5 system. While some articles defined the interoptode distance of 3 cm [27–29, 32], others used distances of 3.2 cm [30]or 2.5 cm [36], or use of varied optode distances (short 1.3 cm, intermediate 2.8 cm, and long 3.8 cm) [34] to observe the same brain region. Furthermore, each study used varying number of sources and detectors, and placement of the probes differed from study to study. Nasi et al. used two sensors and 7 detectors to represent the bilateral M1 regions [34]. Five light sources were used to record hemodynamic change in the left M1, primary sensory cortex (S1), premotor cortex (PM), supplementary motor area (SMA), and prefrontal cortex (PFC), respectively, indicating only one light source used to assess large brain regions [35]. Hada et al. used only two optodes to represent the bilateral primary motor cortices [27]. We believe that the distribution of the minimal number of optodes would likely result in inaccurate representations of brain cortices. These wide variations within as well as between studies make it difficult to conduct proper comparisons and understand changes in hemodynamic activity associated with rTMS, and the results should be interpreted with caution. Third, the large variability in rTMS parameter settings, with regard to type of rTMS, frequency, intensity, duration and stimulation site all made it challenging to determine whether changes in cortical excitability result from differences in parameters. The included studies employed a range of rTMS types and settings, from low frequency to high frequency rTMS, intermittent and prolonged continuous theta-burst stimulation to quadripulse stimulation. Though there is increasing evidence that different rTMS frequencies have differential impact on the brain, more studies with standardized parameter settings and paired sham control are warranted to better understand the impact of rTMS frequency on fNIRS recordings. Additionally, it is essential to address the impact of scalp stimulation on fNIRS measurements. Scalp stimulation induced by rTMS may lead to hemodynamic changes detected by fNIRS, likely arising from TMS-induced muscular stimulation or direct effects on superficial microvasculature [21, 53]. Although various fNIRS techniques, including short-separation detectors, have been proposed to account for these effects by assessing superficial blood flow [53], most of the eligible studies in our review did not utilize such methods. Hence, it is essential for future research to thoroughly investigate and mitigate the influence of scalp stimulation on fNIRS measurements to uphold data accuracy and reliability. Lastly, physiological factors may affect fNIRS measurements. Motion artifacts, rTMS induced facial muscle twitches, aforementioned configuration of fNIRS and rTMS, may potentially introduce transient artifacts into fNIRS data [30]. We advocated these must be closely monitored and controlled to ensure that fNIRS data is accurate.

## Conclusions

This study systematically reviewed the impacts of rTMS on the M1 using fNIRS to measure hemodynamic changes. Despite the diverse study protocol designs and stimulation parameters of rTMS, our analysis revealed disparate patterns of fNIRS measurement among included studies. Nonetheless, our meta-analysis unveiled a consistent trend of increased [HbO] in the contralateral cortices and decreased [HbO] in the ipsilateral cortices following low frequency inhibitory rTMS. These observations underscore the presence of dense mutual interactions between motor cortices across hemispheres, as well as interhemispheric modulation effects. Collectively, these insights provide valuable understanding of the complex neurovascular responses induced by rTMS in the motor cortex, with potential implications for neurological rehabilitation. However, given the high heterogeneity observed the studies, further research is warranted to elucidate the underlying mechanisms of rTMS-induced changes in brain activity during both resting and active conditions.

## Data Availability

No datasets were generated or analysed during the current study.

## Abbreviations

AMT: Active motor threshold
AUC: Area under curve
CBF: Cerebral blood flow
cTBS: Continuous theta-burst stimulation
DLPFC: Dorsolateral prefrontal cortex
EEG: Electroencephalography
fMRI: Functional magnetic resonance imaging
fNIRS: Functional near-infrared spectroscopy
FPC: Frontopolar cortex
Hb: Hemoglobin
HbD: Deoxygenated hemoglobin
HbO: Oxygenated hemoglobin
HK: Anatomical hand knob region
hMHS: Hand motor hotspot region
ISI: Interstimulus interval
iTBS: Intermittent theta-burst stimulation
M1: Primary motor cortex
MEPs: Motor-evoked potentials
MT: Motor threshold
NOS: The Newcastle-Ottawa Scale
PET: Positron emission tomography
PFC: Prefrontal cortex
PM: Premotor cortex
QPS: Quadripulse stimulation
RCT: Randomized controlled trial
RMT: Resting motor threshold
RoB 2.0: Cochrane risk-of-bias tool for randomized trials
rTMS: Repeated transcranial magnetic stimulation
S1: Primary sensory area
SMA: Supplementary Motor Area
